# Relation of burnout to on-call duties among Saudi board residents in Aseer region: A cross-sectional study

**DOI:** 10.1097/MD.0000000000045422

**Published:** 2025-10-31

**Authors:** Hayfa A. AlHefdhi, Syed Esam Mahmood, Zainah G. Alshumrani, Ameerah K. Alzailaie, Sameera A. AL-Aslai, Ahlam M. Alghamdi, Mozoun Dafer Alahmari, Bandar A. Alasmari, Majed M. Al Saleh, Mariyyah Mohammed S. Abdullah, Noura Awad Abusahba

**Affiliations:** a Department of Family and Community Medicine, College of Medicine, King Khalid University, Abha, Saudi Arabia; b Ministry of Health, Abha, Saudi Arabia; c College of Medicine, King Khalid University, Abha, Saudi Arabia; d Pharmacy College, King Khalid University, Abha, Saudi Arabia.

**Keywords:** Maslach, burnout, on-call, post-call, residents, emotional exhaustion, depersonalization, and personal accomplishment, satisfaction

## Abstract

Burnout is a common psychological condition among medical residents, characterized by emotional exhaustion (EE), depersonalization (DP), and reduced personal accomplishment (PA). This study aimed to evaluate the impact of on-call hours on burnout subscales in residents. A cross-sectional study was conducted in the Aseer region, Saudi Arabia, involving 260 residents from various hospitals and residency programs. The Maslach Burnout Inventory was utilized to assess burnout levels. Descriptive statistics summarized the study characteristics and responses. Cronbach alpha was calculated for each subscale for reliability. Mean and standard deviations (mean ± standard deviation) were reported for the subscale scores, while multiple linear regression was used to analyze the relationship between on-call hours and burnout subscales. Mediation analysis, using the Sobel test and bootstrapping, examined the indirect effect of on-call hours on burnout subscales through satisfaction. Among the 260 participants, 52.7% were female, 63.1% aged 27 to 29, and 63.1% were single. The most represented specialties were pediatrics (16.9%) and psychiatry (13.5%), with 29.6% in their first year of residency. Most respondents (90%) reported on-call durations of 17 to 24 hours, but only 36.5% received 3 to 4 hours of post-call rest. High EE, DP, and PA were reported by 72%, 68%, and 37% of residents, respectively. Regression analysis showed that 17 to 24 hours of on-call duty significantly increased EE (6.95; 95% CI: 2.55–11.34, *P* = .002) and DP (6.95; 95% CI: 2.55–11.34, *P* = .002). Post-call rest of 1 to 2 hours reduced EE (−0.8; 95% CI: −15.14 to −0.86, *P* = .028) and increased PA (12.64; 95% CI: 5.36–19.93, *P* = .001). Longer post-call rest (3–6 hours) further improved PA. Mediation analysis revealed that satisfaction partially mediated the effect of on-call hours on PA, reducing burnout (2.32; 95% CI: 0.08–4.55, *P* = .042). The study highlights the high prevalence of burnout among residents in the Aseer region and underscores the need for reforms. Recommendations include limiting on-call shifts to 16 hours and implementing mandatory post-call rest to enhance residents’ well-being and reduce burnout.

## 1. Introduction

Burnout is a prevalent psychological syndrome described as a condition of physical and emotional tiredness brought on by work or caring for others.^[1]^ Dealing in occupations that require interaction with people; therefore, it is common among healthcare providers due to exposure to chronic and work-related stress.^[2]^ Burnout is a multidimensional syndrome consisting of 3 components; emotional exhaustion (EE), which occurs when emotional resources are depleted and physicians feel they can no longer give their best psychologically; depersonalization (DP), which occurs when physicians develop a pessimistic attitude and feelings about their patients; and reduced personal accomplishment (PA), a tendency to negatively evaluate oneself.^[3]^

According to estimates, every third physician experiences at least 1 aspect of medical burnout. This epidemic of burnout is becoming more widespread. Burnout affects medical students in the US at a rate of 31% to 49.6%, physicians at a rate of 30%, and residents at a rate of 50% to 76%. A research by residents of internal medicine at the University of Washington revealed that 76% of them met the Maslach Burnout Inventory (MBI) burnout criteria^[4]^There have also been reports of high levels of burnout in a number of emerging nations, including Malaysia, Saudi Arabia, Lebanon, and Egypt 63.1%.^[4–7]^ On-call duty hours and post-call hours play a fundamental role in burnout in medical residents. Prolonged awake for 24 hours causes cognitive impairment equal to blood alcohol content rise 0.004%.^[8]^ Additionally, a number of research show that residents who deal with these sleep disorders experience increasing feelings of empathy and isolation. In particular, a recent longitudinal study that tracked residents who worked more than 48 hours a week reported that their risk of medical errors, avoidable adverse events, and occupational injuries increased, and at 60 hours or more, their risk doubled^[9]^ Reham Shalaby et al., 2023 reported that the prevalence of burnout among residents was 58.2%, and working more than 80 hours a week significantly increased burnout more than 5 times.^[10]^

Burnout has detrimental consequences on the health system. These include physical and mental health illnesses, depression, suicidal thoughts, workplace disputes, absenteeism, poorer job performance, reduced job commitment, and poor patient care practices. This in turn can eventually force a doctor to leave the medical industry.^[6,11]^

In Saudi Arabia, many studies discussed burnout among residents of Riyadh, Jaddah, and Al Madina. Turki Mohammed Aldrees et al.,2013 reported a high EE in 54%, a high DP in 35%, and a high PA in 33%. Khalid Bawakid et al., 2017 reported that 69.5% or residents had a high EE, Sami A. Aldubai et al., 2019 reported 32% burnout according to the EE subscale only. However, these studies did not focus on the effect of on-call duty hours on burnout, used different cut-points, or reported only for the EE dimension.^[6,12,13]^

In this study, we hypothesized that longer on-call duties hours with less post-call hours among Saudi residents in Aseer region increase burnout represented by MBI-Human Services Survey (MBI-HSS) with its 3 dimensions, EE, DP, and PA. This study aimed to evaluate the impact of on-call hours on burnout subscales in residents.

## 2. Methods

This cross-sectional study took place in Aseer region, Kingdom of Saudi Arabia (KSA). The study population included residents in Aseer region, KSA, agreed to participate in the study, and assigned to on-call duties. Residents currently on medications for psychiatric conditions such as depression or anxiety were excluded from the study to avoid confounding effects on burnout assessment.

Primary outcome: assess the impact of on-call hours on burnout subscales EE, DP, and AP among Saudi residents.

Secondary outcome: assess the burnout subscales predictors in direct and indirect way.

### 2.1. The study tool

In this study, the Maslach Burnout tool was used. This validated tool consisted of 3 subscales EE (nine questions), DP (five questions), and PA (eight questions).^[14]^ The higher the exhaustion or depersonalization scores the higher burnout, the higher the PA score the lower burnout. The cutoff point for each subscale was derived from Viva Combs Thorsen et al., 2011.^[15]^ Questions like demographics age group, gender, marital status, number of children, residency program, residency year, number of on-call hours, and post-call hours were added to the questionnaire.

Operational definitions:^[14]^

EE: Feelings of being emotionally overextended and worn out by one’s work are referred to as emotional tiredness. It is defined by a depletion of emotional resources and represents the stress factor of burnout.
DP: the process of responding to those receiving one’s care or assistance in an impersonal and unfeeling way, which results in a harsh and distant attitude toward those with whom one is dealing. It displays burnout’s interpersonal aspect.
AP: Feelings of competence and effective achievement in one’s work with people are referred to as PAs. In contrast to EE and DP, it is the positive component of burnout and reflects the self-evaluation feature of burnout.

### 2.2. Sample size calculation

A sample 259 was calculated by Raosoft online calculator to achieve power 80% and 0.05 significance level.

### 2.3. Ethical approval

The ethical approval was received from King Khalid University, KSA, ECM#2023-804. Where the data collection process is aligned with the King Khalid University ethical committee.

### 2.4. Data collection procedure

The self-administered structured questionnaire based on MBI-Human Services Survey (MBI-HSS) was applied to 260 residents during the period between 1^st^ April 2023 to 31^st^ March 2024. The questionnaire was applied in Aseer central hospital, Khamis Mushait general hospital, Abha maternity and children hospital, mental health hospital, and Armed force hospital. The tool had been applied to different residency years and programs such as pediatrics, emergency medicine, internal medicine, psychiatry, gynecology, neurology, orthopedic, general surgery, ophthalmology, and radiology.

### 2.5. Statistical analysis

The data was analyzed using SPSS version 24 (Chicago). The study described characteristics and responses using percentages. Cronbach alpha was calculated for each part of the questionnaire, as recommended in the MBI Manual. The scores for each part were reported as average (mean) plus or minus standard deviation (SD).

To see if there were differences in burnout scores based on different characteristics, t-tests and one-way ANOVA tests were used.

To examine how on-call hours affect burnout, multiple linear regression was performed for each burnout aspect. Before running the regression, we checked that the data met necessary assumptions (linearity, normality, independence, and no multicollinearity) using the variance inflation factor. The best model was chosen based on the lowest Akaike information criterion.

Finally, mediation analysis was done to see if job satisfaction explains (mediates) the relationship between on-call hours and burnout. This was tested using Sobel test and bootstrapping, which are good methods for this purpose.

Results were considered statistically significant if the *P*-value was <.05.

## 3. Results

From the 260 respondents, 52.7% were female, 63.1% were in the age group of 27 to 29 years, and 63.1% were single. The most common residency specialties were pediatrics 16.9% and psychiatry 13.5%. Additionally, 29.6% were in their first year of residency. Ninety percent of the respondents reported responding to on-call duties for 17 to 24 hours, and only 36.5% took 3 to 4 hours of post-call rest. Residency program, year, and post-call hours showed significant differences among EE and AP (Table 1).

**Table 1:** Characteristics of residents.

Characteristics	Level	N (%)	EE	DP	AP
Age group	24–26 yr	74 (28.5)	**0.002**	0.338	0.184
	27–29 yr	164 (63.1)			
	30 or above	22 (8.5)			
Gender	Male	123 (47.3)	0.078	0.713	0.073
	Female	137 (52.7)			
Marital status	Single	164 (63.1)	0.42	0.755	0.256
	Married	89 (34.2)			
	Divorced	7 (2.7)			
If married have children?	No	68 (26.2)			
	Yes	43 (16.5)			
Residency program	Pediatrics	44 (16.9)	**<0.001**	0.426	**<0.001**
	Psychiatry	35 (13.5)			
	Radiology	17 (6.5)			
	Gynecology	26 (10.0)			
	Internal medicine	31 (11.9)			
	ENT	23 (8.8)			
	Orthopedic	15 (5.8)			
	Surgery	21 (8.1)			
	Dermatology	20 (7.7)			
	Ophthalmology	21 (8.1)			
	Neurology	2 (0.8)			
	Preventive medicine	1 (0.4)			
	Emergency medicine	2 (0.8)			
	Family planning	1 (0.4)			
	Maxillofacial	1 (0.4)			
Year of residency	R1	77 (29.6)	**0.002**	0.377	**0.015**
	R2	71 (27.3)			
	R3	59 (22.7)			
	R4	46 (17.7)			
	R5 or more	7 (2.7)			
On-calls hours	1–8 h	12 (4.6)	0.065	0.093	**0.002**
	9–16 h	13 (5.0)			
	17–24 h	235 (90.4)			
Post-call hours	None	18 (6.9)	**0.020**	0.230	**<0.001**
	1–2 h	11 (4.2)			
	3–4 h	95 (36.5)			
	5–6 h	83 (31.9)			
	>6	53 (20.4)			

Bold values indicate statistically significant at *P*-value <.001.

DP = depersonalization, EE = emotional exhaustion.

The responses for the exhaustion, depersonalization, and PA subscales are presented in Table 2. The mean ± SD for the exhaustion, depersonalization, and PA subscales are 34.13 ± 0.66, 12.81 ± 0.35, and 34.13 ± 0.96, respectively. The responses indicated that 72% of residents experienced high exhaustion, 16% experienced moderate exhaustion, and 12% experienced low exhaustion. Regarding depersonalization, 68% of respondents reported high levels, 20% reported moderate levels, and 12% reported low levels. Additionally, the PA scores revealed that 37% of respondents had high scores, 23% had moderate scores, and 40% had low scores. The exhaustion and PA subscales demonstrated high reliability, with Cronbach alpha values of 0.911 and 0.900, respectively. In contrast, the depersonalization subscale exhibited poor reliability, with a Cronbach alpha of 0.580 (Table 3).

**Table 2:** The Maslach burnout subscales.

	Never n(%)	A few times a year or less n(%)	Once a month or less n(%)	A few times a month n(%)	Once a week n(%)	A few times a week n(%)	Every day n(%)
I. Emotional Exhaustion							
1. I feel emotionally drained from my work.	5 (1.9)	4 (1.5)	26 (10)	56 (21.5)	57 (21.9)	–	112 (43.1)
2. I feel used up at the end of the workday.	2 (0.8)	10 (3.8)	16 (6.2)	35 (13.5)	44 (16.9)	–	153 (58.8)
3. I feel fatigued when I get up in the morning and have to face another day on the job.	6 (2.3)	12 (4.6)	21 (8.1)	43 (16.5)	51 (19.6)	–	127 (48.8)
6. Working with people all day is really a strain for me.	11 (4.2)	26 (10)	40 (15.4)	74 (28.5)	43 (16.5)	–	66 (25.4)
8. I feel burned out from my work.	4 (1.5)	6 (2.3)	18 (6.9)	25 (9.6)	52 (20)	–	155 (59.6)
13. I feel frustrated by my job.	3 (1.2)	29 (11.2)	31 (11.9)	66 (25.4)	52 (20)	–	79 (30.4)
14. I feel I’m working too hard on my job.	2 (0.8)	12 (4.6)	10 (3.8)	21 (8.1)	45 (17.3)	–	170 (65.4)
16. Working with people directly puts too much stress on me.	18 (6.9)	32 (12.3)	34 (13.1)	76 (29.2)	30 (11.5)	–	70 (26.9)
20. I feel like I’m at the end of my rope.	15 (5.8)	36 (13.8)	36 (13.8)	46 (17.7)	33 (12.7)	–	94 (36.2)
II. Depersonalization							
5. I feel I treat some recipients as if they were impersonal objects.	37 (14.2)	31 (11.9)	31 (11.9)	71 (27.3)	39 (15)	–	51 (19.6)
10. I’ve become more callous toward people since I took this job.	23 (8.8)	30 (11.5)	20 (7.7)	36 (13.8)	49 (13.8)	–	102 (39.2)
11. I worry that this job is hardening me emotionally.	34 (13.1)	67 (25.8)	46 (17.7)	42 (16.2)	31 (11.9)	–	40 (15.4)
15. I don’t really care what happens to some recipients.	173 (66.5)	35 (13.5)	16 (6.2)	17 (6.5)	13 (5)	–	6 (2.3)
22. I feel recipients blame me for some of their problems.	40 (15.4)	48 (18.5)	28 (10.8)	54 (20.8)	42 (16.2)	–	48 (18.5)
III. Personal Accomplishment							
4. I can easily understand how my recipients feel about things.	(1.9)	14 (5.4)	12 (4.6)	24 (9.2)	20 (7.7)	–	185 (71.2)
7. I deal very effectively with the problems of my recipients.	3 (1.2)	12 (4.6)	10 (3.8)	22 (8.5)	39 (15)	–	174 (66.9)
9. I feel I’m positively influencing other people’s lives through my work.	11 (4.2)	13 (5)	18 (6.9)	25 (9.6)	32 (12.3)	–	161 (61.9)
12. I feel very energetic.	27 (10.4)	35 (13.5)	62 (23.8)	71 (27.3)	32 (12.3)	–	33 (12.7)
17. I can easily create a relaxed atmosphere with my recipients.	20 (7.7)	14 (5.4)	21 (8.1)	22 (8.5)	35 (13.5)	–	148 (56.9)
18. I feel exhilarated after working closely with my recipients.	22 (8.5)	22 (8.5)	31 (11.9)	80 (30.8)	38 (14.6)	–	67 (25.8)
19. I have accomplished many worthwhile things in this job.	14 (5.4)	11 (4.2)	18 (6.9)	29 (11.2)	32 (12.3)	–	156 (60)
21. In my work, I deal with emotional problems very calmly.	8 (3.1)	15 (5.8)	17 (6.5)	44 (16.9)	51 (19.6)	–	125 (48.1)

**Table 3:** Mean and reliability of the subscales.

Maslach Burnout subscale	Mean (SD)	Cronbach alpha
Exhaustion	34.13 ± 0.66	0.911
Depersonalization	12.81 ± 0.35	0.580
Accomplishment	34.13 ± 0.96	0.900

SD = standard deviation.

Multiple linear regression analysis for the EE subscale revealed several significant predictors. On-call hours of 17 to 24 hours significantly increased exhaustion 6.95 (95% CI: 2.55–11.34, *P* = .002). Conversely, having 1–2 hours of post-call rest significantly decreased exhaustion −0.8 (95% CI: −15.14 to −0.86, *P* = .028). Residency in family medicine and emergency medicine also significantly increased exhaustion, with coefficients of 29.59 (95% CI: 10.19–48.99, *P* = .003) and 19.61 (95% CI: 4.18–35.04, *P* = .013), respectively. Additional residencies in ENT, gynecology, internal medicine, orthopedics, pediatrics, neurology, psychiatry, ophthalmology, and surgery were also associated with significantly increased exhaustion. Furthermore, being in the fifth year or beyond of residency significantly decreased exhaustion compared to the first year −14.18 (95% CI: −23.21 to −5.15, *P* = .002). While gender, age, and marital status had no significant impact on exhaustion (Table 4).

**Table 4:** Predictors of the exhaustion among residents in Aseer region.

Predictors	Levels	Estimates	CI	*P*-value
(Intercept)		14.73	5.00–24.47	**.003**
How many hours of on call	1–8h	Reference		
	17–24 h	8.93	1.98–15.87	**.012**
	9–16 h	−2.39	−10.98 to 6.21	.585
How many hours of post call	No post call	Reference		
	1–2 h	−8.00	−15.14 to −0.86	**.028**
	3–4 h	−3.78	−8.79 to 1.23	.139
	5–6 h	−3.38	−8.64 to 1.89	.208
	Over 6 h	−2.98	−8.76 to 2.80	.310
Residency	Dermatology	Reference		
	Emergency	19.61	4.18–35.04	**.013**
	ENT	10.95	5.01–16.90	**<.001**
	Family medicine	29.59	10.19–48.99	**.003**
	Gynecology	18.62	13.18–24.05	**<.001**
	Internal Medicine	18.61	12.92–24.30	**<.001**
	Maxillofacial	13.71	−6.57 to 33.99	.184
	Neurology	17.80	4.38–31.21	**.010**
	Ophthalmology	12.02	5.03–19.00	**.001**
	Orthopaedic	17.30	10.46–24.14	**<.001**
	Pediatrics	17.49	12.04–22.94	**<.001**
	Preventive medicine	4.49	−15.15 to 24.14	.653
	Psychiatry	10.68	5.55–15.82	**<.001**
	Radiology	18.58	12.69–24.48	**<.001**
	Surgery	19.56	13.51–25.60	**<.001**
Gender	Male	Reference		
	Female	2.23	−0.31 to 4.76	.085
Age group	24–26 yr	Reference		
	27–29 yr	−1.71	−4.95 to 1.54	.300
	Above 30 yr	−5.15	−10.91 to 0.62	.080
Marital status	Single	Reference		
	Divorced	−5.87	−13.49 to 1.75	.131
	Married	1.10	−1.83 to 4.03	.461
Year of residency	R1	Reference		
	R2	−1.80	−5.14 to 1.54	.289
	R3	−2.40	−6.28 to 1.49	.225
	R4	−3.44	−7.82 to 0.94	.123
	R5 or above	−14.18	−23.21 to −5.15	**.002**

Bold values indicate statistically significant at *P*-value <.001.

CI = confidence interval.

Multiple linear regression demonstrated the DP predictors as on-call hours for 17 to 24 hours increased depersonalizations significantly 6.95 (95% CI: 2.55–11.34,*P* = .002). In addition to the residency specialties, emergency, family, internal medicine, Pediatrics, and surgery significantly increased the depersonalization 9.79 (95% CI: 0.03–19.55, *P* = .049), 14.91 (95% CI: 2.63–27.19, *P* = .018), 5.76 (95% CI: 2.16–9.36, *P* = .002), 3.51 (95% CI:0.06–6.95, *P* = .046), and 4.46 (9%CI: 0.64–8.29, *P* = .002), respectively. While gender, age, year of residency, post-call hours, and marital status had no significant impact on depersonalization (Table 5).

**Table 5:** Predictors of depersonalization among residents of the Aseer region.

	Predictors	Estimates	CI	*P*-value
	(Intercept)	5.35	−0.81 to 11.51	.089
How many hours of on call	9–16 h	Reference		
	17–24 h	6.95	2.55–11.34	**.002**
	9–16 h	4.00	−1.44 to 9.43	.149
How many hours of post call	No post call	Reference		
	1–2 hrs	−2.16	−6.68 to 2.36	.347
	3–4 hrs	0.30	−2.87 to 3.47	.854
	5–6 hrs	−0.98	−4.31 to 2.35	.561
	Over 6 hrs	0.44	−3.22 to 4.09	.814
Residency	Dermatology	Reference		
	Emergency	9.79	0.03–19.55	**.049**
	ENT	3.64	−0.12 to 7.40	.058
	Family medicine	14.91	2.63–27.19	**.018**
	Gynecology	3.45	0.01–6.89	.050
	Internal Medicine	5.76	2.16–9.36	**.002**
	maxillofacial	1.19	−11.64 to 14.02	.855
	Neurology	−1.71	−10.20 to 6.77	.691
	Ophthalmology	−0.56	−4.98 to 3.86	.805
	Orthopaedic	3.37	−0.96 to 7.70	.127
	Pediatrics	3.51	0.06–6.95	**.046**
	Preventive medicine	6.73	−5.70 to 19.16	.287
	Psychiatry	2.19	−1.06 to 5.44	.185
	Radiology	1.55	−2.18 to 5.28	.414
	Surgery	4.46	0.64–8.29	**.022**
Gender	Male	Reference		
	Female	−0.78	−2.38 to 0.83	.341
Age group	24–26 yr	Reference		
	27–29 yr	−0.06	−2.12 to 1.99	.951
	Above 30 yr	−1.37	−5.02 to 2.27	.458
Marital status	Single	Reference		
	Divorced	−2.40	−7.22 to 2.42	.328
	Married	−0.82	−2.67 to 1.04	.386
Year of residency	R1	Reference		
	R2	−0.91	−3.03 to 1.20	.395
	R3	0.05	−2.41 to 2.50	.970
	R4	0.86	−1.91 to 3.64	.539
	R5 or above	−3.53	−9.24 to 2.18	.224

Bold values indicate statistically significant at *P*-value <.001.

CI = confidence interval.

Multiple linear regression illustrated the predictors of PA as post-call hours 1 to 2hrs, 3 to 4hr, and 5–6 hrs significantly increased the accomplishment, lower the burnout, 12.64 (95% CI: 5.36–19.93,*P* = .001), 12.32 (95% CI: 7.20–17.43,*P* < .001), and 10.75 (95% CI: 5.37–16.12,*P* < .001), respectively. Where the residency specialties such as family medicine, gynecology, neurology, pediatrics, and psychiatry significantly decreased the accomplishment, increased burnout, −21.94 (95CI: −41.75 to −2.13, *P* = .030), −7.68 (95% CI: 13.23 to −2.13, *P* = .007), −18.26 (95% CI: −31.96 to −4.57, *P* = .009), −14.40 (95% CI: −19.96 to −8.83, *P* < .001), and −5.80 (95% CI: −11.04 to −0.56, *P* = .030), respectively. While gender, age, Year of residency, on-call hours, and marital status had no significant impact on the accomplishment (Table 6).

**Table 6:** Predictors of PA among residents of the Aseer region.

	Predictors	Estimates	CI	*P*-value
	(Intercept)	24.94	15.00–34.88	**<.001**
How many hours of on call	9–16 h	Reference		
	17–24 h	6.42	−0.67 to 13.51	.076
	9–16 h	1.27	−7.50 to 10.05	.776
How many hours of post call		Reference		
	1–2 h	12.64	5.36–19.93	**.001**
	3–4 h	12.32	7.20–17.43	**<.001**
	5–6 h	10.75	5.37–16.12	**<.001**
	Over 6 h	4.35	−1.56 to 10.25	.148
Residency	Dermatology	Reference		
	Emergency	4.00	−11.76 to 19.75	.618
	ENT	−1.07	−7.14 to 5.00	.729
	Family medicine	−21.94	−41.75 to −2.13	**.030**
	Gynecology	−7.68	−13.23 to −2.13	**.007**
	Internal Medicine	−3.09	−8.89 to 2.72	.296
	Maxillofacial	12.69	−8.02 to 33.39	.228
	Neurology	−18.26	−31.96 to −4.57	**.009**
	Ophthalmology	3.11	−4.02 to 10.25	.391
	Orthopaedic	−4.97	−11.95 to 2.02	.163
	Pediatrics	−14.40	−19.96 to −8.83	**<.001**
	Preventive medicine	−18.09	−38.15 to 1.96	.077
	Psychiatry	−5.80	−11.04 to −0.56	**.030**
	Radiology	−5.68	−11.70 to 0.34	.064
	Surgery	−1.02	−7.20 to 5.15	.744
Gender	Male	Reference		
	Female	−1.66	−4.25 to 0.93	.208
Age group	24–26 years	Reference		
	27–29 years	1.63	−1.68 to 4.94	.333
	Above 30 years	3.35	−2.54 to 9.23	.264
Marital status	Single	Reference		
	Divorced	−3.08	−10.87 to 4.70	.436
	Married	0.15	−2.84 to 3.15	.919
Year of residency	R1	Reference		
	R2	−0.79	−4.20 to 2.62	.649
	R3	2.22	−1.74 to 6.19	.271
	R4	1.89	−2.58 to 6.37	.405
	R5 or above	−8.63	−17.85 to 0.58	.066

Bold values indicate statistically significant at *P*-value <.001.

CI = confidence interval, PA = personal accomplishment.

### 3.1. Mediation analysis

**Figure 1.**
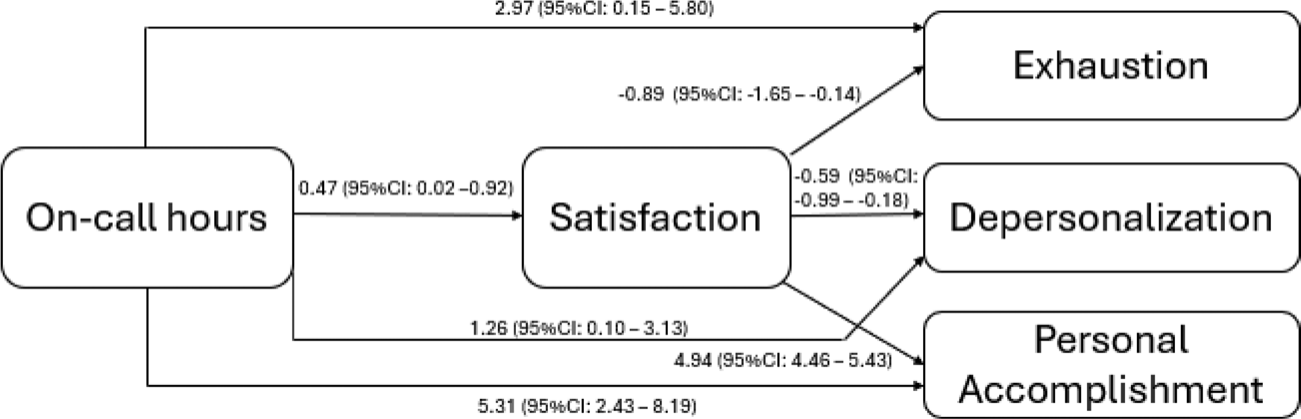
Mediation model illustrated the direct and indirect effects of on-call hours on burnout subscales.

To determine if the satisfaction had a mediation effect, Sobel test was used. The mean of satisfaction was calculated to be 4.73 ± 1.73. The results indicate that the direct effect of on-call hours was significant on the exhaustion, depersonalization, and PA was 2.97 (95% CI: 0.15–5.80, *P* = .039), 1.62 (95% CI: 0.10–3.13,*P* = .037), and 5.31 (95% CI: 2.43–8.19, *P* < .001). The direct of on-call hours on satisfaction was significant 0.47 (95% CI: 0.02–0.92, *P* = .043) (Fig. 1). The indirect effect of on-call hours on exhaustion mediated by satisfaction nonsignificantly decreased the exhaustion by −0.41 (95% CI: −0.95–0.11, *P* = .125). The indirect effect of on-call hours on depersonalization mediated by satisfaction was nonsignificantly decreased DP −0.27 (95% CI: −0.60–0.04, *P* = .096). The indirect effect of on-call hours on PA mediated by satisfaction significantly increased by 2.32 (95% CI: 0.08–4.55, *P* = .042) which lower burnout (Fig. 1).

## 4. Discussion

This study illustrated the burnout among Saudi residents in the Aseer region using a validated tool MBI-HSS for medical personnel. EE subscale with mean score 34.13 ± 0.66 reported in this study to be high in 72%,while DP with mean 12.81 ± 0.35 reported high score in 68%, and PA with mean 34.13 ± 0.96 reported high score in 37%. The burnout reported among physicians in the developed countries like Switzerland, Italy and France ranged from 30 to 50%, likely because of high patient expectations and demands.^[16]^ Meanwhile, the prevalence of burnout reported in developing countries was higher. Alenezi et al., 2022 hours reported high EE at 57.51% and high DP as 36.62%.^[17]^ Many Saudi Arabian studies reported burnout between 25.2% and 70%. Khalid Bawakid et al, 2017 in Jeddah, KSA reported lower subscale scores EE 69.5%, DP 26%, and PA 12.2% but they used different cut-point for the subscales, however the mean ± SD was lower for each subscale EE, DP, and AP than in our study 11.60 ± 4.70, 5.66 ± 5.20, and 14.44 ± 3.66, respectively.^[12,13,17]^

In this study, call hours of 17 to 24 hours increased EE significantly 8.93 (95% CI: 1.98–15.87, *P* = .012) and post-call hours of 1 to 2 hours decreased EE significantly −8.00 (95% CI: −15.14 to −0.86, *P* = .028). The fifth year of residency had significant effect to decrease EE (*P* = .002). Moreover, most residency programs increased EE significantly compared to others except those with dermatology, preventive medicine, and maxillofacial programs. The DP subscale increased significantly with on-call hours 17 to 24 hours 6.95 (95% CI: 2.55–11.34, *P* = .002) and with some residency programs than others such as emergency, internal medicine, pediatrics, and surgery. For the AP subscale, our results illustrated that 1 to 6 post-call hours increased the AP score, lower burnout, and some residency programs such as family medicine, gynecology, pediatric, psychiatry, and neurology decreased the AP score, which increases the burnout subscale.

Sami A. R. Aldubai et al., 2019 in Al Madina, KSA reported only for the EE subscale considering it as a good reflection of burnout, where the mean EE of residents was 22.5 ± 12.8, and only 32.0% showed high EE.^[13]^ This could be explained that in Jaddah the number of residents was higher than in Aseer, which could decrease the burden of on-call duties and burnout. Working in shifts *P* = .026 and residency year *P* = .039 founded to significantly increase EE.

Khalid Bawakid et al., 2017 reported that patient violence is a significant predictor that increases burnout among physicians 0.36 (95% CI: 2.93–5.55, *P* < .001).^[12]^ Suffering from back pain (OR = 2.1, 95% CI 1.2–3.8, *P* = .01), having sleep deprivation (OR = 2.2, 95% CI: 1.2–3.8, *P* = .009), being a resident physician/surgeon (OR = 4.9, 95% CI: 1.7–14.2, *P* = .004), and having a negative effect of practice on family life (OR = 2.1, 95% CI: 1.1–3.9, *P* = .02) were reported in Turki Mohammed Aldrees et al., 2022 reported working more than 80 hours/week (OR = 16.437; 95% CI: 2.059–131.225), being dissatisfied (OR = 22.28; 95% CI: 1.75–283.27) were significant predictors for burnout among resident in Canada.^[10]^

Increased on-call hours that resulted in sleep deprivation were associated with increased depression and burnout. Many theories explaining the link between sleep deprivation and burnout propose chronic energy depletion or activation of the hypothalamic-pituitary-adrenal axis along with elevated levels of physical stress as the underlying mechanisms.^[18]^

Shu Feng et al.,2023, a cohort study of residents of ophthalmology at University Washington recommended mandatory post-call hours to improve post-call sleep and lower EE, DP and PA.^[19]^

Our findings regarding the mediation model illustrated the nonsignificant effect of satisfaction as a mediator, on EE or DP. However, satisfaction significantly diluted the effect of on-call hours on AP through indirect effect 2.32 (95% CI: 0.08–4.55, *P* = .042), as when the PA subscale increased, the burnout decreased. Hui Zhang et al., 2021 highlighted the significant indirect effect of EE on the satisfaction through both optimism and work engagement 0.03 (95% CI: 0.01–0.05).^[20]^

This study had some limitations. First, it was a cross-sectional study that lacks causal inferences and directionality. Second, the satisfaction measure by 1 aspect of job satisfaction; we recommend that future studies include different aspects of job performance and to be longitudinal studies. Finally, the medical history of the residents was not included in this study, which could affect the burnout subscales. However, this study had some strengths, to our knowledge, it was the first study in Aseer that evaluated burnout among residents and focused on specific call and post-call hours. This first study used the mediation analysis to assess the satisfaction effect on burnout.

## 5. Conclusions and recommendations

The study highlighted a high prevalence of burnout among Saudi residents in the Aseer region, emphasizing the need for greater attention to prevent its consequences. It recommended implementing mandatory post-call hours and limiting on-call shifts to no more than 16 hours to improve residents’ quality of life and reduce burnout.

## Author contributions

**Conceptualization:** Hayfa A. AlHefdhi.

**Data curation:** Mozoun Dafer Alahmari, Bandar A Alasmari, Mariyyah Mohammed S Abdullah, Noura Awad Abusahba.

**Investigation:** Hayfa A. AlHefdhi, Zainah G. Alshumrani, Ameerah K. Alzailaie, Sameera A. AL-Aslai, Ahlam M. Alghamdi, Mozoun Dafer Alahmari, Bandar A Alasmari, Majed M.Al Saleh, Mariyyah Mohammed S Abdullah, Noura Awad Abusahba.

**Methodology:** Hayfa A. AlHefdhi, Mozoun Dafer Alahmari, Bandar A Alasmari, Majed M.Al Saleh, Mariyyah Mohammed S Abdullah, Noura Awad Abusahba.

**Project administration:** Hayfa A. AlHefdhi.

**Supervision:** Hayfa A. AlHefdhi.

**Validation:** Syed Esam Mahmood.

**Visualization:** Syed Esam Mahmood.

**Writing – original draft:** Hayfa A. AlHefdhi.

**Writing – review & editing:** Syed Esam Mahmood.
